# Mitochondria-targeting graphene oxide nanocomposites for fluorescence imaging-guided synergistic phototherapy of drug-resistant osteosarcoma

**DOI:** 10.1186/s12951-021-00831-6

**Published:** 2021-03-19

**Authors:** Wei-Nan Zeng, Qiu-Ping Yu, Duan Wang, Jun-Li Liu, Qing-Jun Yang, Zong-Ke Zhou, Yi-Ping Zeng

**Affiliations:** 1 Department of Orthopedics, West China Hospital/West China School of Medicine, Sichuan University, Chengdu, 610041 China; 2 Department of Orthopedics, Chongqing General Hospital, University of Chinese Academy of Sciences, Chongqing, 400014 China; 3 Health Management Center, West China Hospital/West China School of Medicine, Sichuan University, Chengdu, 610041 China

**Keywords:** Synergistic phototherapy, Mitochondria-targeting, Drug-resistant osteosarcoma, Graphene oxide, Single-laser activation

## Abstract

**Background:**

Osteosarcoma (OS) is the most common primary malignant bone tumor occurring in children and young adults. Drug-resistant osteosarcoma often results in chemotherapy failure. Therefore, new treatments aimed at novel therapeutic targets are urgently needed for the treatment of drug-resistant osteosarcoma. Mitochondria-targeted phototherapy, i.e., synergistic photodynamic/photothermal therapy, has emerged as a highly promising strategy for treating drug-resistant tumors. This study proposed a new nano-drug delivery system based on near-infrared imaging and multifunctional graphene, which can target mitochondria and show synergistic phototherapy, with preferential accumulation in tumors.

**Methods and results:**

Based on our previous study, (4-carboxybutyl) triphenyl phosphonium bromide (TPP), a mitochondria-targeting ligand, was conjugated to indocyanine green (ICG)-loaded, polyethylenimine-modified PEGylated nanographene oxide sheets (TPP-PPG@ICG) to promote mitochondrial accumulation after cellular internalization. Thereafter, exposure to a single dose of near-infrared irradiation enabled synergistic photodynamic and photothermal therapy, which simultaneously inhibited adenosine triphosphate synthesis and mitochondrial function. Induction of intrinsic apoptosis assisted in surmounting drug resistance and caused tumor cell death. After fluorescence imaging-guided synergistic phototherapy, the mitochondria-targeting, multifunctional graphene-based, drug-delivery system showed highly selective anticancer efficiency in vitro and in vivo, resulting in marked inhibition of tumor progression without noticeable toxicity in mice bearing doxorubicin-resistant MG63 tumor cells.

**Conclusion:**

The mitochondria-targeting TPP-PPG@ICG nanocomposite constitutes a new class of nanomedicine for fluorescence imaging-guided synergistic phototherapy and shows promise for treating drug-resistant osteosarcoma.

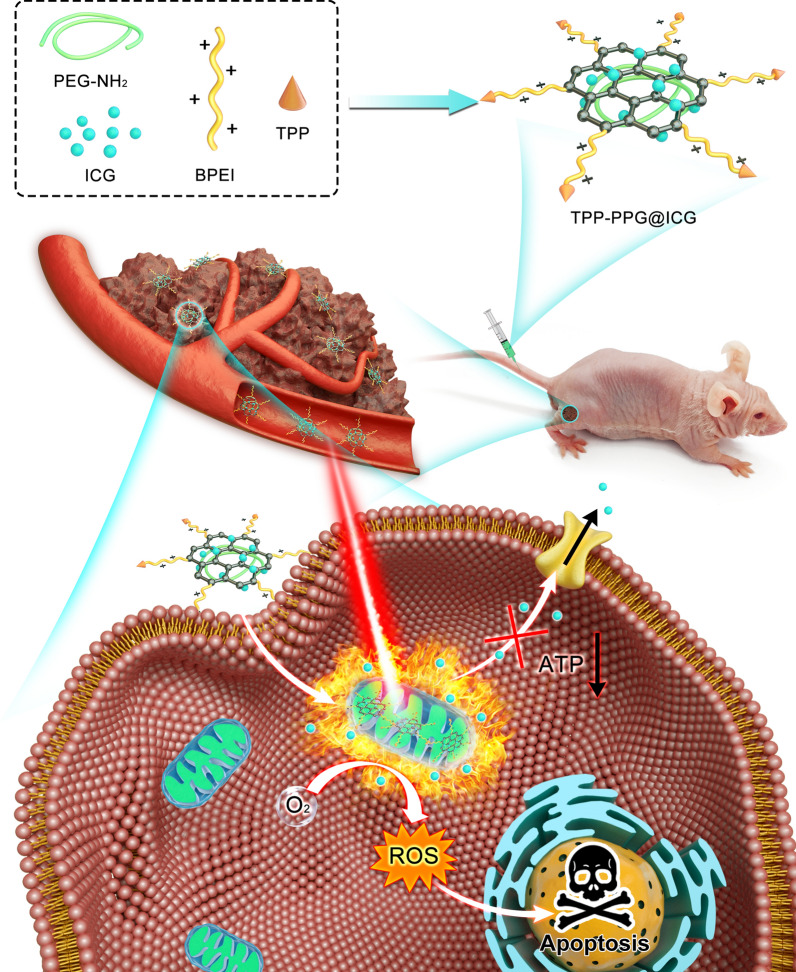

**Supplementary Information:**

The online version contains supplementary material available at 10.1186/s12951-021-00831-6.

## Introduction

Osteosarcoma (OS) is the most common primary malignant bone tumor occurring in children and young adults. Currently, the gold standard for OS treatment consists of polychemotherapy, followed by radical excision of tumor and metastases, and subsequent polychemotherapy. The 5-year survival rate is 70% in patients with non-metastatic disease or ~ 20% in patients with metastatic or recurrent disease [1, 2]. Unfortunately, this treatment achieves disease control in ≤ 60% of patients with OS. Moreover, the prognosis of OS has not improved in recent decades [3, 4]. Drug resistance represents the major limitation of current therapies. Such resistance arises from overexpression of adenosine triphosphate (ATP)-binding cassette transporters, such as P-glycoprotein (P-gP), which expulses anticancer drugs and the selection for drug-resistant cell clones after treatment [5]. These drug-resistant clones can ultimately lead to tumor recurrence or metastatic progression [6]. Therefore, new treatments aimed at novel therapeutic targets are urgently needed.

Recently, mitochondria have received extensive attention as effective targets for tumor treatment. Mitochondria play vital roles in various biological processes, such as initiation of the intrinsic-apoptosis pathway [7–9]. Mitochondrial alterations are hallmarks of oncogenesis, tumor progression, angiogenesis, and chemotherapeutic resistance [10]. During mitochondrial apoptosis, cytochrome c is released from the mitochondria, which subsequently activates the caspase cascade. Hence, targeting mitochondria holds tremendous therapeutic potential for eradicating cancer [10–13].

Synergistic phototherapy, represented by photodynamic therapy (PDT) and photothermal therapy (PTT), can be used to treat drug-resistant tumors with better treatment outcomes than chemotherapy. Synergistic phototherapy is a non-invasive, highly selective, and effective approach with low systemic toxicity for cancer treatment [14]. In phototherapy, light is administered, absorbed by a photosensitizer (PS) or photothermal agent, and converted into reactive oxygen species (ROS) or local hyperthermia, leading to tumor cell death [15]. The rational combination of PDT and PTT may synergistically improve therapeutic outcomes and efficacies for cancer treatment [16]. However, due to spectral mismatches between PDT and PTT agents in the near-infrared (NIR) region, sequential irradiations by two different lasers are required to activate both PDT and PTT, which complicates the treatment process and prolongs the treatment time. In addition, it is difficult to precisely align two laser beams at the same position. Furthermore, some intrinsic barriers such as a short lifetime (10–320 ns) and limited diffusion radius (10–55 nm) of ROS decrease the therapeutic outcome of PDT [17]. Thus, using a single NIR laser to activate synergistic phototherapy offers promise for generating ROS in cellular organelles that are vulnerable to ROS.

Among the cellular organelles, the mitochondrion is the most sensitive to ROS and hyperthermia-mediated damages that can rapidly perturb mitochondrial functions, change the mitochondrial membrane potential, reduce ATP production, and induce tumor cell apoptosis [18–21]. Thus, selective generation of ROS and hyperthermia via phototherapy in the mitochondria may considerably improve their therapeutic efficacy. Furthermore, combined hyperthermia and suppressed ATP production due to ROS-mediated mitochondrial dysfunction could be another effective strategy for surmounting drug resistance [12]. Therefore, mitochondria-targeted synergistic phototherapy may serve as an alternative strategy in drug-resistant OS therapy.

Thus, selectively and efficiently enhancing the mitochondrial accumulation of PS and photothermal agents, which can be efficiently and simultaneously triggered by a single NIR laser that is accurately focused on tumor regions, and sufficiently converting the optical energy to ROS and hyperthermia, are vitally important. Hence, during phototherapy, it is necessary to outline the tumor margin in real-time to demarcate it from the normal tissue and focus the laser beam accurately on the tumor regions. Fluorescence (FL) imaging in the NIR region (700–900 nm) can be used to monitor in real-time the dynamic distribution of PS and photothermal agents in vivo, and to detect tumors and tumor margins [22]. Furthermore, to enhance the treatment efficiency, reduce the number of operation steps and the treatment time, and completely use the synergistic effect of PDT and PTT, the PS and the photothermal agents must simultaneously delivered to mitochondria as composite nanostructures and triggered using a single NIR laser [23, 24]. However, synergistic phototherapy with a single laser strongly depends on overlap between the optical absorption spectra of the PS and photothermal agents. This special requirement and complex synthesis processes may limit their combined use. However, a great demand exists for developing simple, yet highly effective nanocomposites to coordinate PTT with PDT.

**Scheme 1.**
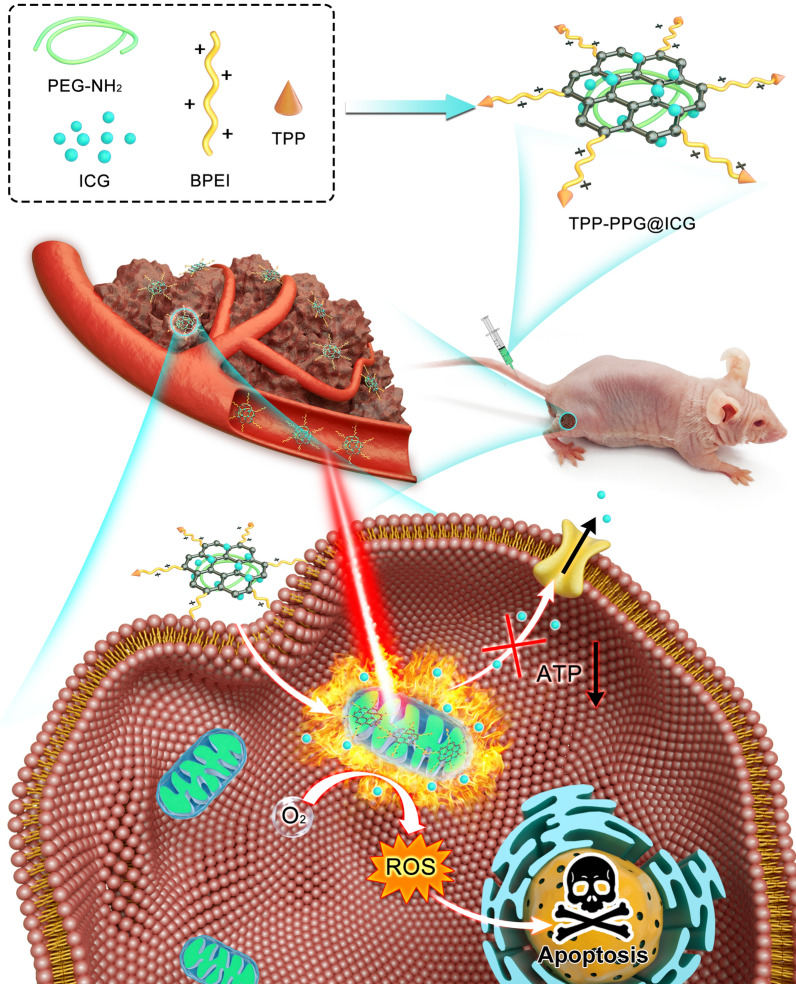
Schematic illustration showing a TPP-PPG@ICG nanocomposite targeting a mitochondrion for synergistic phototherapy with a single laser. The PS agent (ICG) was grafted onto the PEG- and BPEI-functionalized photothermal agent (NGO) to obtain TPP-PPG@ICG. After cellular internalization, TPP-PPG@ICG accumulated in mitochondria, induced mitochondria-related intrinsic apoptosis, surmounted drug resistance, and enhanced the antitumor efficacy after laser irradiation at 808 nm

Indocyanine green (ICG), the only NIR contrast agent approved by the FDA for clinical use, can not only be used for NIR FL imaging, but can also convert optical energy to ROS and local hyperthermia for synergistic phototherapy [25]. Thus, ICG can be considered an ideal theranostic platform for tumor treatment [26]. However, several disadvantages have prevented the application of ICG in tumor theranostics, including: (i) short half-life in the circulation; (ii) poor photo-stability; (iii) the possibility of unidirectional expulsion by P-gP; (iv) the lack of cancer-specific accumulation for targeted treatment; (v) concentration-dependent aggregation, and (vi) limited efficacy in cancer phototherapy and the need to combine it with other treatments to improve therapeutic outcomes [27]. Hence, we designed a new nanocomposite based on a polyethylenimine-modified PEGylated nanographene oxide (PPG), as we described previously [28–31]. NGO is a planar nanomaterial with an extremely high loading capacity for therapeutic agents, high cellular uptake, and a good photothermal effect, which can enhance the phototherapy of ICG. Furthermore, graphene oxide and ICG can be optimally excited with a single-wavelength NIR laser at 808 nm, which is also suitable for NGO for PTT. Functionalization with polyethylene glycol (PEG), referred to hereafter as GO-PEG, exhibited high delivery efficiency and the controllable release of proteins, gene medicines, bioimaging agents, chemotherapeutics, and anticancer drugs. GO-PEG has a good biological safety profile and causes no significant side effects in vitro or in vivo; in addition, it can be gradually excreted over time [32–34]. Besides, cationic polymer polyethyleneimine (PEI)-modified GO showing excellent stability in physiological solutions and electropositivity which may promote nanocomposite intracellular delivery [35]. Otherwise, the amido functional groups of PEI located at the edge of PPG are abundant for further functionalization. Subsequently, (4-Carboxybutyl) triphenyl phosphonium bromide (TPP), a mitochondria-targeting ligand, was conjugated with PPG via covalent interaction between the amido functional groups of PEI and the carboxy group of TPP to yield the TPP-PPG@ICG nanocomposite [11, 36]. After intravenous administration, the resulting nanocomposite accumulated in tumors due to the enhanced-permeability-and-retention (EPR) effect, targeted the mitochondria, and killed cancer cells via phototherapy in situ upon laser irradiation. Ultimately, this led to robust antitumor efficacy with reduced systemic adverse effects (Scheme 1).

## Materials and methods

### Materials

TPP ((4-Carboxybutyl)triphenyl phosphonium bromide), NaN_3_, *N*-(3-dimethylamino propyl-*N*′-ethylcarbodiimide) hydrochloride (EDC·HCl), and *N*-hydroxysuccinimide (NHS) were obtained from Sigma-Aldrich (Saint Louis, MO, USA). FBS, penicillin–streptomycin, and trypsin were purchased from Gibco (Grand Island, NY, USA). The ROS Kit (containing DCFH-DA), singlet oxygen sensor green (SOSG), MitoTracker, MitoSOX Red, calcein AM, Propidium iodide (PI), and the J-aggregate-forming lipophilic cation (JC-1) were purchased from Molecular Probes (Eugene, OR, USA). The ATP Determination kit (A22066) and cell lysis buffer (16,189) were purchased from Invitrogen (Carlsbad, CA, USA). DMEM medium was purchased from HyClone (Logan, UT, USA). Matrigel and the Annexin V-FITC Apoptosis Detection Kit were purchased from BD Biosciences (San Diego, CA, USA). ICG and the Cell Counting Kit-8 (CCK-8) were purchased from Dojindo (Kumamoto, Japan). Dialysis ultrafiltration tubes and bags with a 10-kDa molecular weight cut-off were purchased from Millipore, Inc. (Billerica, MA, USA). All chemicals and reagents were of analytical grade.

### Cells, animals, and tumor xenografts

The human OS cell line MG63 and the doxorubicin-resistant OS cell line (MG63/Dox) were purchased from the American Type Culture Collection (USA). Both cell lines were grown as monolayers in DMEM medium supplemented with 10% FBS and 1% penicillin–streptomycin at 37 °C in a 5% CO_2_ humidified atmosphere.

Athymic male nude mice (aged 6 weeks, weighing 18–22 g) were obtained from the Laboratory Animal Center of the Chongqing General Hospital (Chongqing, China) and were housed in individual vented cages under specific pathogen-free conditions with a 12 h day/12 h night cycle; food and water were provided ad libitum. All animal protocols were reviewed and approved by the Institutional Animal Care and Use Committee of the Chongqing General Hospital. MG63/Dox cells (approximately 1 × 10^7^) were resuspended in 100 μL Matrigel and subcutaneously implanted into the right flanks of different mice. Tumor-bearing mice were used for in vivo imaging and for phototherapy when the tumor volume reached 60 mm^3^.

### Synthesis of NGO-PEG-BPEI (PPG), TPP-PPG and TPP-PPG@ICG

NGO was prepared according to the modified-Hummer’s method, starting with the oxidization of graphite sheets, followed by ultrasonication [37]. NGO-PEG (PG) and the NGO-PEG-BPEI (PPG) were prepared according to our previously described method [28–31]. To conjugate TPP with PPG, 5 mg of TPP was dissolved in 4 mL water and activated using EDC·HCl (15 mg) and NHS (15 mg) for 15 min at room temperature. Subsequently, 4 mL of PPG (1.0 mg/mL) solution was added to the reaction mixture and magnetically stirred at room temperature for 24 h. Finally, excess TPP was removed via filtration through a 10-kDa filter (Millipore, Inc.) and washed repeatedly with double-distilled water to obtain PPG-TPP (NGO-PEG equivalent, 0.5 mg/mL).

To synthesize TPP-PPG@ICG, ICG (7.74 mg) was dissolved in 1 mL of anhydrous dimethyl sulfoxide as a stock solution (10 mM) for further use. Two hundred microliters of ICG (10 mM) and 1.8 mL of TPP-PPG (0.5 mg/mL) were mixed and stirred for 24 h at room temperature. Then, the whole system was dialyzed against distilled water for 24 h (molecular weight cut-off: 10 kDa). The final product (TPP-PPG@ICG) was freeze-dried and stored below 4 °C for further use.

### Characterization

Fourier transform infrared spectroscopy (FT-IR) spectra were obtained for TPP-PPG@ICG using a Nicolet 6700 spectrometer (Thermo Scientific) to confirm that ICG was loaded onto the NGO. An atomic force microscope (AFM) instrument (Bruker Dimension Icon) was used to characterize the size and thickness of TPP-PPG@ICG. The optical properties of TPP-PPG, ICG, and TPP-PPG@ICG were characterized using an Ultraviolet–visible–infrared (UV–vis–NIR) spectrometer in 50% isopropyl alcohol (UV-3600 Scanning Spectrophotometer, Shimadzu, Japan). The hydrodynamic size of TPP-PPG and TPP-PPG@ICG in water were tested by DLS using a ZetaPALS analyzer (Brookhaven Instruments, Holtsville, NY, USA).

Nanoparticle stability was tested as follows: TPP-PPG or TPP-PPG@ICG was incubated with PBS, DMEM medium, or PBS + 10% fetal bovine serum (FBS), and the mixtures were monitored for the appearance of precipitates after direct centrifugation at 5000 rpm min^−1^ for 10min. Additionally, the TPP-PPG@ICG were incubated with PBS and PBS + 10% FBS for 0, 4, 12 and 24 h, then the stability of nanoparticles was tested by DLS using a ZetaPALS analyzer (Brookhaven Instruments, Holtsville, NY, USA).

The release of ICG from TPP-PPG@ICG was studied by adding the material to acidic (pH = 5.0) or alkalescent (pH = 7.4) PBS at 37 ℃ or 43 ℃, respectively. To determine the release kinetics of ICG from TPP-PPG@ICG, PBS was collected after centrifugation and replaced with the same volume of PBS at each sampling time. The amounts of ICG released from TPP-PPG were evaluated using a UV–vis–NIR spectrometer for loading-efficiency measurements. To determine the ICG loading on TPP-PPG, TPP-PPG@ICG solution was diluted in 5 mL ethyl acetate/ethanol (9:1, v/v) and sonicated for 30 min to completely release ICG. ICG levels were determined by measuring the UV–vis–NIR absorption spectra. ICG loading was defined as follows: ICG content (%, w/w) = (ICG weight in TPP-PPG@ICG/TPP-PPG weight) × 100%. All the measurements were performed in triplicate.

### Single oxygen detection

The generation of singlet oxygen (^1^O_2_) was evaluated using SOSG. Typically, solutions containing ICG, TPP-PPG, and TPP-PPG@ICG (ICG concentration = 10 μM) were mixed with SOSG, which was dissolved in water containing 2% methanol to a final concentration of 1 mM. Then, the mixture was immediately irradiated with a laser at 808 nm for 5 min (energy density: 0.6 W/cm^2^). The emission peak of SOSG at 530 nm was obtained by excitation with a light source at 494 nm, and the data were quantified for singlet oxygen generation.

### Measurements of photothermal performance

One milliliter each of PBS, ICG, TPP-PPG, and TPP-PPG@ICG in aqueous solution was placed in a quartz cell and irradiated at 808 nm with a power density of 0.6 W/cm^2^ for 10 min at pre-designed time intervals (0 min, 2.5 min, 5.0 min, 7.5 min, and 10.0 min). The temperature was measured using an infrared thermometer.

### Cellular uptake and intracellular localization

MG63/Dox cells were seeded in 6-well plates at a density of 1 × 10^5^ cells/well. After overnight incubation, TPP-PPG@ICG was added at a final concentration of 10 μM. After 1 h, 4 h, 8 h, 12 h, or 24 h of incubation, the cells were washed thrice with PBS, trypsinized, resuspended in medium, and harvested for analysis using flow cytometry using a FACSVerse instrument (BD Biosciences). The mean FL intensity of 1 × 10^4^ cells was recorded for each sample.

To study subcellular localization, MG63/Dox cells (1 × 10^5^ cells per mL) were exposed to TPP-PPG@ICG (ICG concentration = 10 μM) on 35-mm glass-bottom dishes for 24 h. After rinsing thrice with PBS, the cells were treated with MitoTracker for 10 min to stain the mitochondria. Then, the cells were washed thrice with PBS before capturing images using a confocal laser-scanning microscope (CLSM).

### In vitro analysis

MG63/Dox cells were seeded in 96-well plates (5 × 10^3^ cells/well in 100 µL) and incubated for 24 h. Then, ICG, TPP-PPG, PPG@ICG, or TPP-PPG@ICG was added to various concentrations of ICG. Subsequently, the cells in the non-irradiated groups were rinsed with PBS and incubated for another 24 h without laser irradiation. The cells in the irradiated groups were exposed to 808-nm laser light at 0.6 W/cm^2^ for 5 min and incubated for another 24 h. Then, cell viabilities were assessed by performing CCK-8 assays. In addition, cell viabilities were also assessed in the presence of NaN_3_ or at low temperature to verify the participation and individual therapeutic efficacies of PDT and PTT. MG63/Dox cells were seeded in 96-well plates (5 × 10^3^ cells/well in 100 µL) and incubated for 24 h. Then, TPP-PPG@ICG was added at various concentrations. NaN_3_ was added to cell culture medium at 100 mM to quench singlet oxygen molecules and thereby block the effect of PDT. Cells were irradiated at 4 °C to maintain a constant temperature and nullify the effect of PTT. Negative-control groups were treated with the laser only or with TPP-PPG@ICG without irradiation. The cells in the irradiated groups were exposed to 808-nm laser light at 0.6 W/cm^2^ for 5 min and incubated for another 24 h. Subsequently, cell viabilities were assessed by performing CCK-8 assays.

Calcein AM/PI co-staining was also performed to assess the synergistic phototherapeutic effect of TPP-PPG@ICG. For visualization, MG63/Dox cells (1 × 10^5^ cells per well) were first seeded in 6-well plates and incubated overnight. Then, the cells were treated with equivalent dosages of PBS (−), TPP-PPG@ICG (−), ICG (+), TPP-PPG (+), PPG@ICG (+), or TPP-PPG@ICG (+) (NGO 20 μg/mL; ICG 15 μM) for 24 h, where “(−)” indicates that no irradiation was applied, and “(+)” indicates that the cells were irradiated at 0.6 W/cm^2^ for 5 min. After incubation for another 24 h, the cells were incubated with calcein AM (to visualize live cells) and PI (to visualize dead/late apoptotic cells), according to the manufacturer’s suggested protocol.

### Detection of apoptosis and intracellular ROS

MG63/Dox cells were seeded overnight in 6-well plates (2 × 10^5^ cells per well) and then treated for 24 h with equivalent dosages of PBS (−), TPP-PPG@ICG (−), ICG (+), TPP-PPG (+), PPG@ICG (+), or TPP-PPG@ICG (+), where “(−)” indicates that no irradiation was applied, and “(+)” indicates that the cells were irradiated at 0.6 W/cm^2^ for 5 min) (NGO equivalent, 20 μg/mL; ICG equivalent, 15 μM). The cells were collected at 6 h post-laser irradiation after careful trypsinization and low-speed centrifugation, followed by washing twice with PBS. The collected cells were resuspended in 100 μL binding buffer and stained with 2 μL annexin V-FITC and 2 μL PI for 15 min at room temperature in the dark. After staining, the cells were collected via low-speed centrifugation, washed twice with PBS, and diluted with 400 μL binding buffer for flow cytometric analysis using an Epics XL-MCL instrument (Beckman Coulter).

The 2′,7′-dichlorodihydrofluorescein diacetate (DCFH-DA) Kit was used to detect the intracellular ROS generation. MG63/Dox cells were seeded overnight in 24-well plates (1 × 10^5^ cells per well) and then treated with equivalent dosages of PBS (−), TPP-PPG@ICG (−), ICG (+), TPP-PPG (+), PPG@ICG (+), or TPP-PPG@ICG (+) (NGO equivalent, 20 μg/mL; ICG equivalent, 15 μM) for 24 h, where “(−)” indicates that no irradiation was applied and “(+)” indicates that the cells were irradiated at 0.6 W/cm^2^ for 5 min. After irradiation, the cells were promptly washed with PBS and incubated with 10 μM DCFH-DA at 37 °C for 30 min. ROS FL signals were evaluated using a DMLRB inverted FL microscope (Leica).

### Detecting the mitochondrial membrane potential

Changes in the mitochondrial membrane potential were measured using JC-1 and imaged using a CLSM. Briefly, MG63/Dox cells (1 × 10^5^ cells per well) were incubated with equivalent dosages of PBS (−), TPP-PPG@ICG (−), ICG (+), TPP-PPG (+), PPG@ICG (+), or TPP-PPG@ICG (+) (NGO equivalent, 20 μg/mL; ICG equivalent, 15 μM), where “(−)” indicates that no irradiation was applied and “(+)” indicates cases where the cells were irradiated for 24 h. The irradiated groups were then illuminated using a laser at 808 nm (0.6 W/cm^2^, 5 min) and incubated for another 24 h. The non-irradiated groups were incubated for 48 h under the same conditions. Then, the cells were promptly washed with PBS and incubated with 5 mM JC-1 at 37 °C for 30 min. The cells were rinsed again with PBS and analyzed using a CLSM.

### Detection mitochondrial superoxide levels

Mitochondrial superoxide generation was assessed by measuring MitoSOX FL using a CLSM. Briefly, MG63/Dox cells were seeded in glass-bottom 35-mm plates overnight and then treated with equivalent dosages of PBS (−), TPP-PPG@ICG (−), ICG (+), TPP-PPG (+), PPG@ICG (+), or TPP-PPG@ICG (+) (NGO equivalent, 20 μg/mL; ICG equivalent, 15 μM), where “(−)” indicates that no irradiation was applied and “(+)” indicates cases where the cells were irradiated at 0.6 W/cm^2^ for 5 min. After treatment, the cells were washed twice with PBS, incubated with 5 µM MitoSOX for 10 min, washed twice with PBS, and analyzed using a CLSM.

### ATP determination assay

A standard curve was generated for a series of ATP concentrations, using an ATP Determination Kit. MG63/Dox cells were seeded in 96-well plates (1 × 10^4^ cells per well) in 100 μL of DMEM and incubated for 24 h prior to performing ATP-determination assays. Then, the cells were treated with equivalent dosages of PBS (−), TPP-PPG@ICG (−), ICG (+), TPP-PPG (+), PPG@ICG (+), or TPP-PPG@ICG (+) (NGO equivalent, 20 μg/mL; ICG equivalent, 15 μM), where “(−)” indicates that no irradiation was applied and “(+)” indicates cases where the cells were irradiated at 0.6 W/cm^2^ for 5 min. After 6 h of incubation, the culture medium was removed, and the cells were treated with lysis buffer. Next, the reagents of the ATP Determination Kit were added to the lysed cells, and a plate reader was used to measure the luminescence in order to calculate each ATP concentration. Each experiment was repeated thrice, and mean values were calculated.

### NIR FL and thermal imaging

In vitro thermal imaging of the PBS blank, ICG, and TPP-PPG@ICG under irradiation (808 nm, 0.6 W/cm^2^, 5 or 10 min) was conducted using an infrared thermal-imaging camera (Ti32, Fluke, USA). TPP-PPG@ICG was injected via the tail vein (0.5 mg/kg) into athymic nude mice bearing MG63/Dox tumor xenografts to evaluate the tumor-targeting ability. In vivo NIR imaging was taken from 0 to 48 h after injection with an in vivo NIR Imaging System (Kodak). Thermal imaging of blank PBS and TPP-PPG@ICG under irradiation (808 nm, 0.6 W/cm^2^, 5 min) was recorded by an infrared thermal imaging camera (Ti32, Fluke, USA). All the sets and imaging conditions were the same as those of the reported method [38]. The major organs that were excised were imaged after sacrificing the nude mice that were intravenously administered with TPP-PPG@ICG at 24 h post-injection. Regional body temperatures and infrared thermographic maps were obtained using a Ti27 infrared thermal imaging camera (Fluke).

### Combined in vivo treatment with PDT and PTT

When the tumor volume reached 60 mm^3^, the tumor-bearing mice were randomly divided into six groups, with five mice per group. These groups included the PBS-treated, non-irradiated group (control group); the TPP-PPG@ICG-treated, non-irradiated group; the ICG-treated, irradiated group; the TPP-PPG-treated, irradiated group; the PPG@ICG-treated, irradiated group; and the TPP-PPG@ICG-treated, irradiated group. Equivalent dosages of the therapeutic agents were injected intravenously (ICG equivalent, 750 μM). In addition, for the irradiation groups, laser irradiation at 808 nm (0.6 W/cm^2^, 5 min) was applied at the tumor site. Variations in tumor volumes and body weights in each group were monitored every 3 days for up to 15 days to evaluate the therapeutic effects. Then, the animals were euthanized on day 15. The tumors and major organs (the heart, liver, spleen, lungs, and kidneys) were collected. H&E and TUNEL staining of tumor sections from different groups were performed, and the stained samples were observed under a bright-field microscope (Olympus).

### In vivo toxicity assessment

The 200 μL TPP-PPG@ICG (ICG equivalent, 750 μM) was injected intravenously into five healthy male nude mice. Another five nude mice injected with normal saline were selected as the control group. Then, at days 30, the blood was collected from each mouse for the blood chemistry test and complete blood panel analysis.

### Statistical analysis

All statistical analyses were performed with SPSS 13.0 software. Data were presented as mean ± standard deviation. The significance of the data is analyzed according to a Student’s t-test: *P < 0.01.

## Results and discussion

### Synthesis and characterization of TPP-PPG@ICG

Based on the modified-Hummer’s method, NGO preparation was initiated by the oxidization of graphite sheets, which was followed by ultrasonication [37]. Then, PG was prepared via a ring-opening nucleophilic addition reaction between the amine groups of the amino-terminated PEG (average molecular weight: 5000 Da) and the epoxy groups of NGO, according to our previously developed method [28–31]. This PEGylation method generated individual small NGO nanosheets with carboxyl functional groups at their edges, which were subsequently covalently conjugated with BPEI (average molecular weight: 1800 Da). Well-dispersed PPG was prepared and functionalized with abundant amine groups using this modification. Subsequently, TPP (a mitochondria-targeting ligand) was conjugated to the surface of the nanocomposite (TPP-PPG). Finally, ICG was absorbed onto TPP-PPG via π–π stacking and hydrophobic interactions to obtain the TPP-PPG@ICG nanocomposite. Then, the TPP-PPG@ICG nanocomposite was purified via ultrafiltration and washed with 50% isopropyl alcohol repeatedly until FL in the filtrate could not be detected by NIR [39–43].

**Fig. 1:**
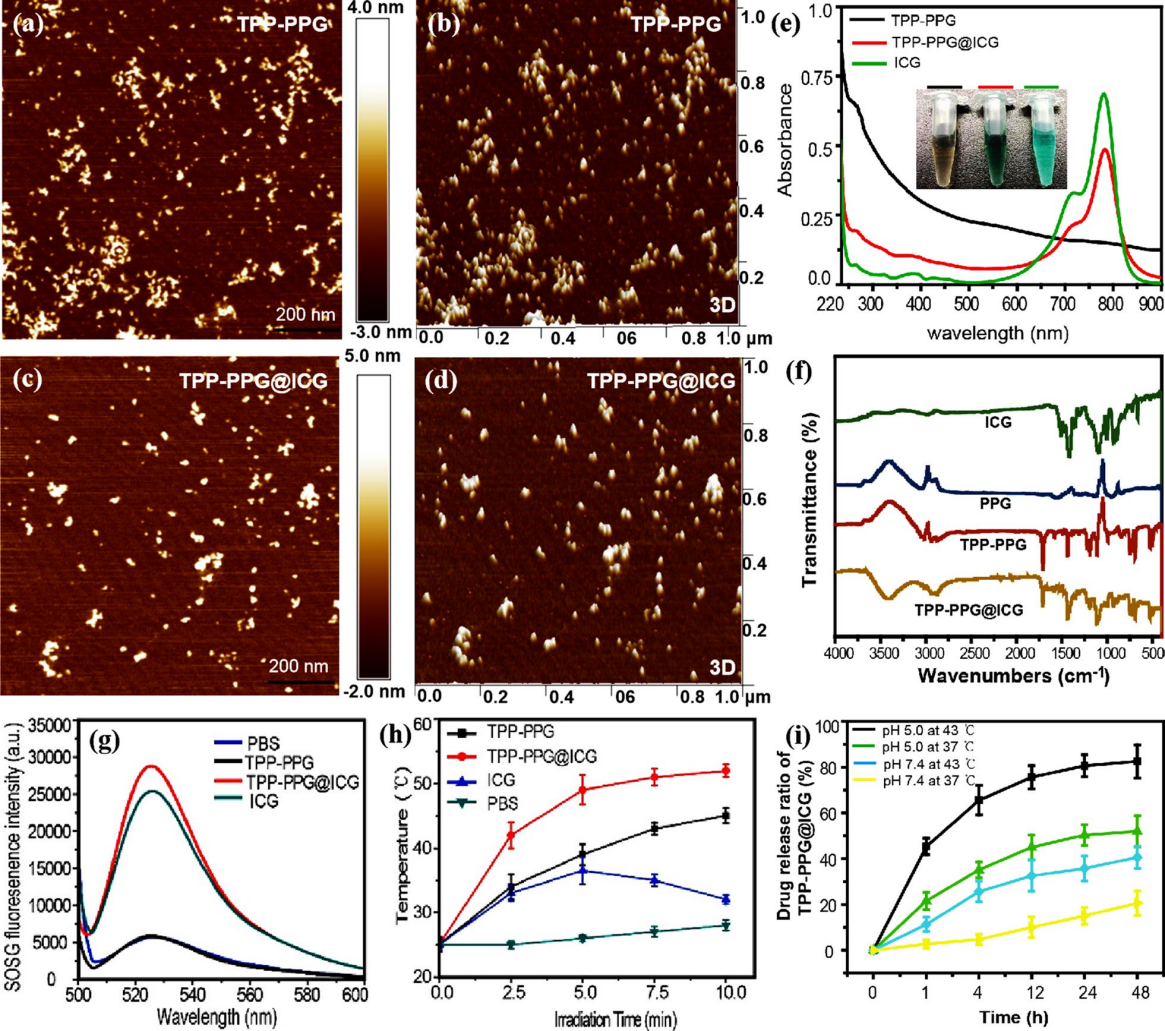
Characterization and physicochemical properties of TPP-PPG@ICG. Atomic force microscopy (AFM)-based morphological characterizations of **a**–**d** TPP-PPG and TPP-PPG@ICG. **e** UV–vis absorption spectra the indicated materials. The inset shows photographs of aqueous solutions of TPP-PPG, TPP-PPG@ICG, and ICG. **f** FT-IR spectra of ICG, PPG, TTP-PPG, and TPP-PPG@ICG. **g** FL intensity of SOSG at 530 nm in PBS, TPP-PPG, TPP-PPG@ICG, and ICG after laser irradiation at 808 nm (0.6 W/cm^2^, 5 min). **h** Heating curves of PBS, ICG, TPP-PPG@ICG, and TPP-PPG during a 10-min irradiation. **i** ICG-release rate of TPP-PPG@ICG in acidic (pH = 5.0) and alkalescent PBS (pH = 7.4) at 37 °C and 43 °C

The size, thickness, and morphology of the TPP-PPG@ICG nanocomposite were characterized using AFM. As shown in Fig. 1a–d, the TPP-PPG@ICG nanocomposite formed small sheets measuring approximately 20–50 nm on one side (∼ 1 nm thickness). Successful ICG loading was evident in the UV–vis–NIR absorbance and FT-IR spectra of the aqueous dispersions (Fig. 1e, f). The results showed that TPP-PPG@ICG exhibited characteristic absorbance peaks of PPG (281 nm) and a characteristic broad absorption of TPP-PPG@ICG in the NIR region, ranging from 700 to 800 nm. These data confirmed the successful loading of ICG onto PPG. Meanwhile, the hydrodynamic diameters of TPP-PPG and TPP-PPG@ICG in water were detected by a DLS test. Depending on the DLS value, the mean diameters of the TPP-PPG and TPP-PPG@ICG in water were approximately 103 nm and 115 nm, respectively (Additional file 1: Fig. S1).

The stabilities of TPP-PPG@ICG in phosphate-buffered saline (PBS, pH 7.4), Dulbecco’s modified Eagle’s medium (DMEM), or PBS +10% fetal bovine serum (FBS) were compared (Additional file 1: Fig. S2a). After direct centrifugation at 5000 rpm min^−1^ for 10 min, no precipitation was observed. Then, the ultrafiltration tubes (10 kda) were used to collect the filtrate. As shown in Additional file 1: Fig. S2a, the pale green was observed in the filtrate except for those derived from media, suggesting a little ICG was filtered out. The results showed that TPP-PPG loaded with ICG via π–π stacking and hydrophobic interactions was stable, which is important for the ability of PPG to serve as a carrier to deliver PS agents or drugs into tumor cells via the EPR effect after intravenous injection [44, 45]. In order to research how much of the loaded ICG is released after incubation with PBS, DMEM and PBS + 10%FBS, the UV–vis peak at 790 nm was recorded to determine the changes before/after incubation. The release of the ICG was calculated after subtraction of absorbance contributed by TPP-PPG. As displayed in Additional file 1: Fig. S2b, it was discovered that the released rate of ICG from TPP-PPG@ICG was 17.2%, 15.2% and 18.5% in PBS, DMEM and PBS + 10%FBS, respectively. Noticeably, there is a small amount of loss because some ICG was adhered to the ultrafiltration tube (Additional file 1: Fig. S2a). Hence, the results showed that the only little ICG release from TPP-PPG@ICG after incubation with PBS, DMEM and PBS + 10%FBS, suggesting the TPP-PPG@ICG were stable in the physiological solutions.

To further demonstrate the stability of TPP-PPG@ICG in PBS and PBS + 10% FBS, we performed the DLS test. As exhibited in Additional file 1: Fig. S3, the hydrodynamic size of the TPP-PPG@ICG in PBS and PBS + 10% FBS (approximately 140–160 nm) were slightly increased compared with those in water (approximately 115 nm) and exhibited no significant changes over the course of 24 h, suggesting that the nanoparticles are highly stable in physiological solution. This result was consistent with that of centrifugation shown in Additional file 1: Fig. S2. Moreover, an ICG-loading efficiency of 0% to 30.4% was achieved by adjusting the ICG: TPP-PPG weight ratio (Additional file 1: Fig. S4), which is calculated according to the standard curve for ICG is shown in Additional file 1: Fig. S5.

The photodynamic property of TPP-PPG@ICG was assessed by determining its ability to generate singlet oxygen (^1^O_2_) based on the FL signal of singlet oxygen sensor green (SOSG), a highly sensitive ^1^O_2_ indicator. SOSG was added to different solutions of PBS or ICG (15 μM), TPP-PPG@ICG (equivalent to 15 μM ICG), or TPP-PPG (NGO 20 μg/mL) in PBS, and irradiated for 5 min with a laser at 808 nm (power density: 0.6 W/cm^2^). Considerably higher SOSG FL intensity at 530 nm was observed in the ICG and TPP-PPG@ICG solutions than in the blank PBS and TPP-PPG solutions (Fig. 1g), indicating that TPP-PPG@ICG could act as a PS for PDT applications. The photothermal effect of TPP-PPG@ICG was evaluated by determining the temperatures of different samples after exposure to laser irradiation at 808 nm. The results showed that the temperature of the PBS blank increased slowly from 25 to 26 °C within 5 min of irradiation (Fig. 1h). However, the temperature of TPP-PPG@ICG, increased sharply from 25 to 48 °C with a 35.8% photothermal conversion efficiency, which could lead to irreversible damage to tumor cells. It is also noteworthy that the photothermal-generation abilities of TPP-PPG and TPP-PPG@ICG increased proportionally with the duration of laser irradiation, whereas the photothermal-generation ability of ICG first increased and then decreased.

To demonstrate hyperthermia and pH trigged drug release, the drug release from the TPP-PPG@ICG were comparatively investigated in 37 °C and 43 °C while the pH value at 5.0 and 7.4. As shown in Fig. 1i, the ICG release from TPP-PPG@ICG at 37 °C and pH 7.4 was very low, only about 15% of the total loaded ICG was released after 48 h. However, the release of ICG was significantly accelerated at 43 °C and pH 5.0, reaching a cumulative release of about 42.5% of total loaded drug in 1 h and 79.2% in 24 h. This may be due to that the π–π stacking interaction between ICG and TPP-PPG became weaker at acidic microenvironment and hyperthermia, which could not efficiently load the drug. Collectively, the release of the loaded ICG from the nanoparticles was found to follow a complex temperature and pH value dependent relationship. Therefore, if the TPP-PPG@ICG is located in acidic microenvironment and high temperature, ICG could release from TPP-PPG to have a superior synergistic therapeutic effect.

**Fig. 2:**
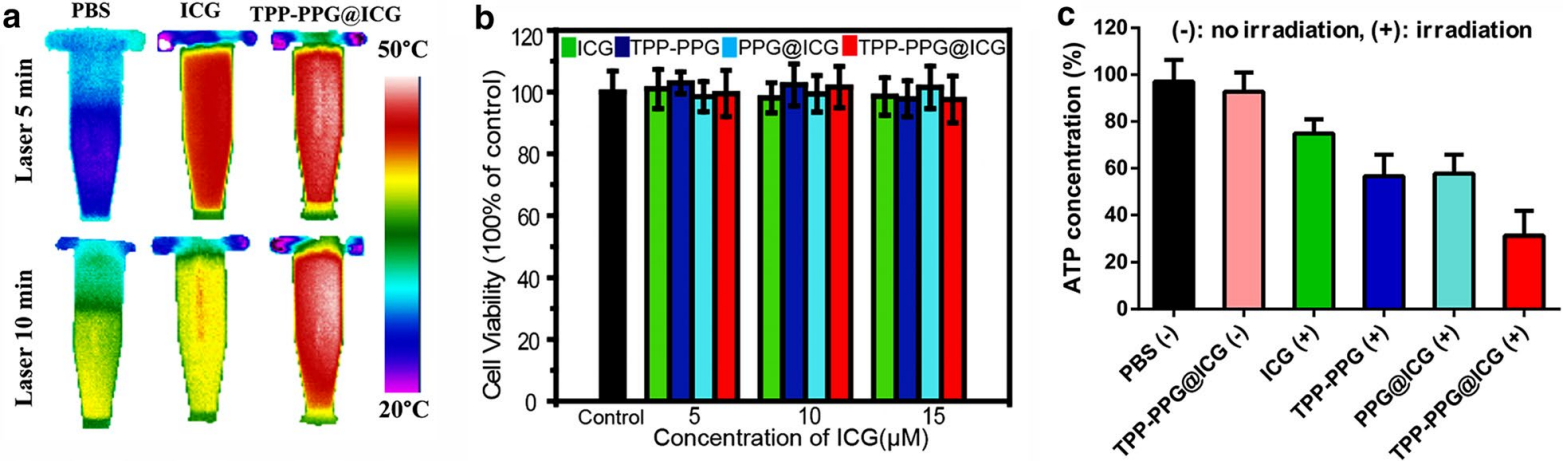
**a** Thermal images of PBS, ICG, and TPP-PPG@ICG during a 5-min irradiation (upper row) or a 10-min irradiation (bottom row). **b** Cell Counting Kit-8 (CCK-8)-based viability assay performed with doxorubicin-resistant MG63 cells (MG63/Dox cells) treated with ICG, TPP-PPG, PPG@ICG, or TPP-PPG@ICG, without laser irradiation. **c** Relative intracellular ATP concentrations in the MG63/Dox cells after incubation with PBS, TPP-PPG@ICG, ICG, TPP-PPG, or PPG@ICG for 6 h. Cells were treated with or without the NIR laser (808 nm, 0.6 W/cm^2^) for 5 min, and then their intracellular ATP concentrations were measured

The IR thermal images (Fig. 2a) of blank PBS, ICG, and TPP-PPG@ICG in vitro during 5 and 10 min of irradiation were recorded, and the results also indicated that TPP-PPG@ICG continuously generated greater heat than the PBS blank or ICG, whereas ICG showed attenuated photothermal generation during long-time irradiation. These results demonstrated that the ICG can undergo irreversible photochemical degradation upon NIR laser irradiation and that nanographene sheets can effectively avoid this intrinsic defect. These results demonstrated that TPP-PPG@ICG possessed stronger ROS- and photothermal-generation abilities than TPP-PPG or ICG alone. Thus, combining both can result in synergistically improved phototherapy.

### In vitro synergistic phototherapeutic effects and cellular uptake of TPP-PPG@ICG

To verify the drug resistance of the human OS cell line, different concentrations of doxorubicin (0.5–2.5 μg/mL) were added to the MG63 and the MG63/Dox cells in culture. The CCK-8 assay results indicated that the viability of the MG63/Dox cells remained above 90%, whereas the viability of the MG63 cells was reduced to 24.9% when the doxorubicin concentration reached 2.5 μg/mL (Additional file 1: Fig. S6).

**Fig. 3:**
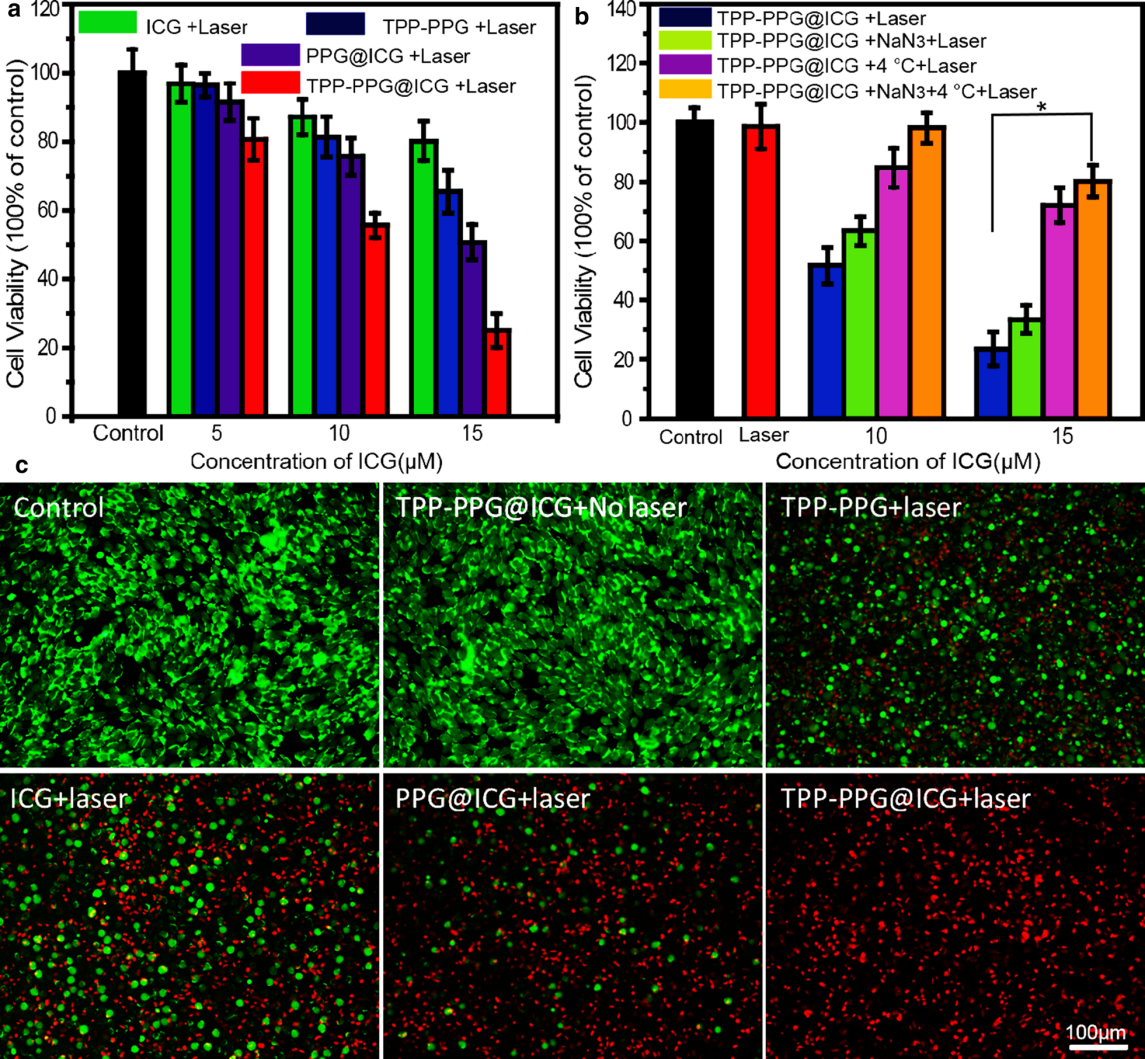
Synergistic in vitro phototherapeutic effects of TPP-PPG@ICG and laser irradiation on MG63/Dox cells. **a** CCK-8 viability assay of MG63/Dox cells treated with ICG, TPP-PPG, PPG@ICG, or TPP-PPG@ICG 24 h after laser irradiation at 808 nm. **b** CCK-8 viability assays with MG63/Dox cells exposed to TPP-PPG@ICG at 24 h post-laser irradiation at 808 nm, under conditions that inhibited PDT (with NaN_3_ which quenches ^1^O_2_) or PTT (with a low temperature to inhibit photothermal generation). **c** FL images of calcein AM + PI co-stained MG63/Dox cells after treatment with PBS, TPP-PPG, ICG, PPG@ICG, or TPP-PPG@ICG (ICG equivalent, 15 μM) at 24 h post-laser irradiation, or treatment with TPP-PPG@ICG without laser irradiation. P values in **a** and **b** were calculated using student’s t-test (*p < 0.01)

An in vitro phototherapeutic study of TPP-PPG@ICG was performed with the MG63/Dox cancer cells. Cell-viability assays were conducted at 24 h post-irradiation (0.6 W/cm^2^, 5 min) at an ICG-equivalent concentration ranging from 5 to 15 μM. The results of the CCK-8 assays indicated that a negligible change occurred in the cell viabilities of non-irradiated groups treated with ICG, TPP-PPG, PPG@ICG, or TPP-PPG@ICG (Fig. 2b). The cell viability of all groups was > 95%, which indicated good biological compatibility. However, the cell viability of the irradiated groups gradually decreased with increasing concentrations of ICG, TPP-PPG, PPG@ICG, or TPP-PPG@ICG (Fig. 3a). In particular, the phototoxicity of 15 μM TPP-PPG@ICG was markedly higher than that of individual treatment with ICG, TPP-PPG, or PPG@ICG treatments. The mitochondria-targeting TPP-PPG@ICG nanocomposite showed stronger phototoxicity than the non-mitochondria-targeting nanocomposite, PPG@ICG, which indicated that the efficacy of phototherapy was improved with mitochondria-targeted treatment. In order to research whether PDT or PTT works for the phototherapy effect, the cytotoxic effect of individual PDT and PTT was studied using sodium azide (NaN_3_, which blocks the cytotoxic effect of ROS) and at 4 °C (to block the effect of PTT). As exhibited in Fig. 3b, the viabilities of the cells treated with TPP-PPG@ICG were 51.6% and 24.9% after treatment with 10 μM or 15 μM ICG, respectively. Interestingly, the cell-killing efficiency of TPP-PPG@ICG was markedly inhibited by treatment with 100 mM NaN_3_ (PTT alone), incubation at 4 °C (PDT alone) or the two treatments together, and the most inhibition effect was observed when the cells treated with 100 mM NaN_3_ and incubation at 4 °C. Hence, PTT or PDT treatment alone did not completely kill the cells, as evident from the cell-viability assays. Otherwise, it is noteworthy that the killing efficacy of PTT alone was higher than that of PDT alone. Collectively, the results suggested that both PDT and PTT were collectively responsible for the cytotoxicity of TPP-PPG@ICG, with the effect of PTT representing the major cytotoxic mechanism.

Effective phototoxicity was further confirmed by performing calcein AM/propidium iodide (PI) double-staining assays with MG63/Dox cells after a 24-h incubation. Red and green FL from calcein AM and PI corresponded to dead and live cells, respectively. Cells in the control group and the group treated with TPP-PPG@ICG alone displayed green FL, suggesting that these treatments were not toxic to MG63/Dox cells. In the TPP-PPG + laser, ICG + laser, and PPG@ICG + laser groups, some cells were killed and displayed red FL. The TPP-PPG@ICG + laser group induced most of cells death and exhibited intense red FL. These data further illustrate the synergistic therapeutic effect of mitochondria-targeting PDT and PTT (Fig. 3c).

**Fig. 4:**
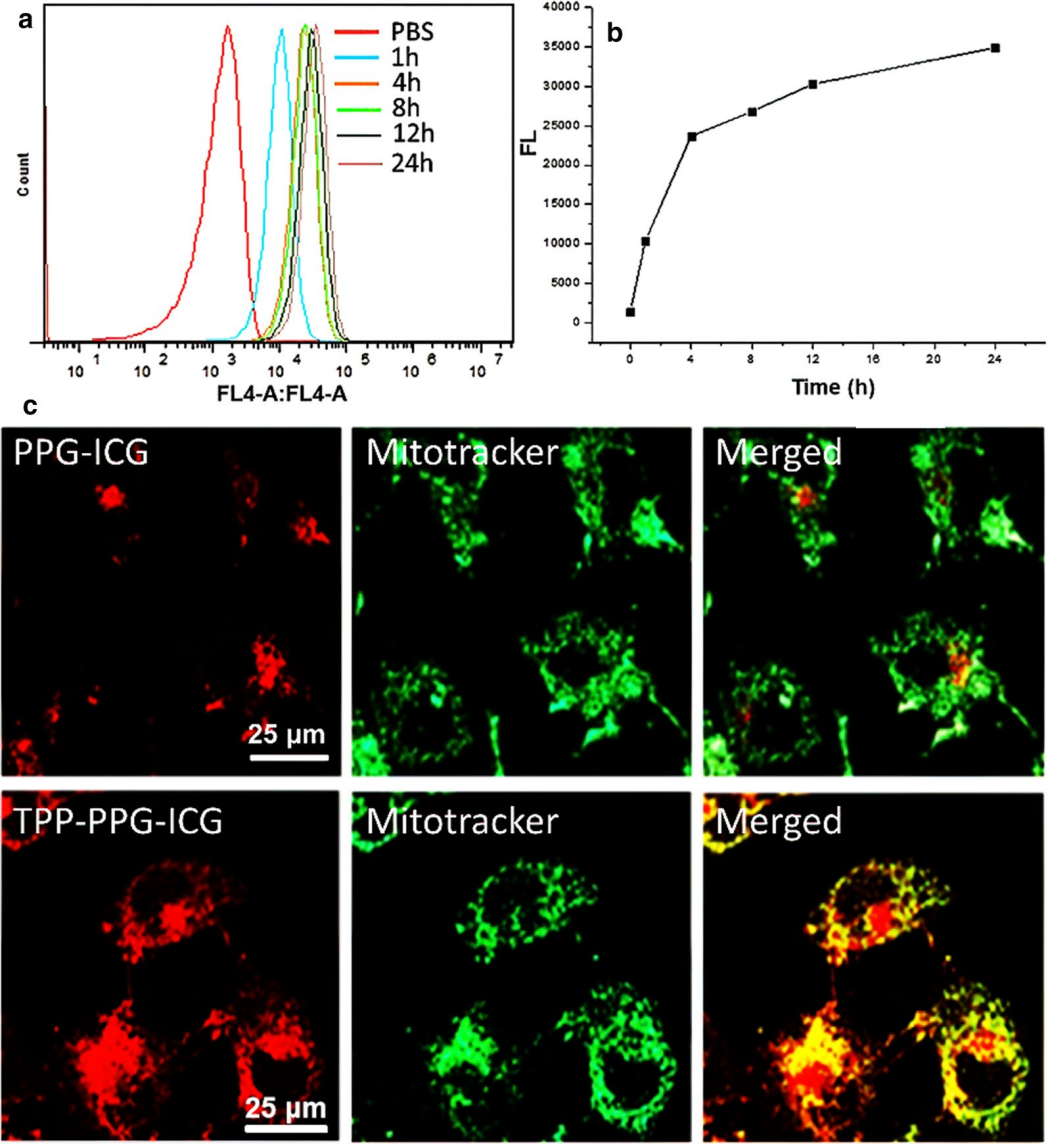
Cellular uptake and subcellular localization. **a** The cellular uptake of TPP-PPG@ICG was measured by flow cytometry. **b** The mean FL intensity of MG63/Dox cells (n = 10,000 cells) was determined by flow cytometric analysis. **c** FL-confocal microscopic images of MG63/Dox cells co-treated with MitoTracker and either PPG@ICG or TPP-PPG@ICG are shown

Effective cellular uptake is particularly important for enhancing the penetration and retention of nano-anticancer drugs in tumor regions. To study intracellular, MG63/Dox cells were incubated with PBS or 15 μM TPP-PPG@ICG for 1 h, 4 h, 8 h, 12 h, and 24 h, after which they were analyzed by flow cytometry. The mean FL intensity of MG63/Dox cells treated with TPP-PPG@ICG increased in a time-dependent manner, showing a rapid uptake in the first 4 h after incubation and saturation at 12 h (Fig. 4a, b). These findings were consistent with the results of our previous studies, which showed that PEG- and BPEI-functionalized nanomaterials showed good cellular uptake ability when used as a delivery carrier [28–31]. The different uptake rates may also explain the higher phototoxicity of TPP-PPG@ICG than that of ICG at the same concentration. To examine subcellular localization, MG63/Dox cells were co-stained with the MitoTracker after incubation with TPP-PPG@ICG or PPG@ICG. FL-confocal microscopy images indicated that TPP-PPG@ICG accumulated in the mitochondria of tumor cells (Fig. 4c). This result confirmed the efficiency of mitochondrial targeting by TPP-PPG@ICG.

### Cellular mechanism of synergistic phototherapy

**Fig. 5:**
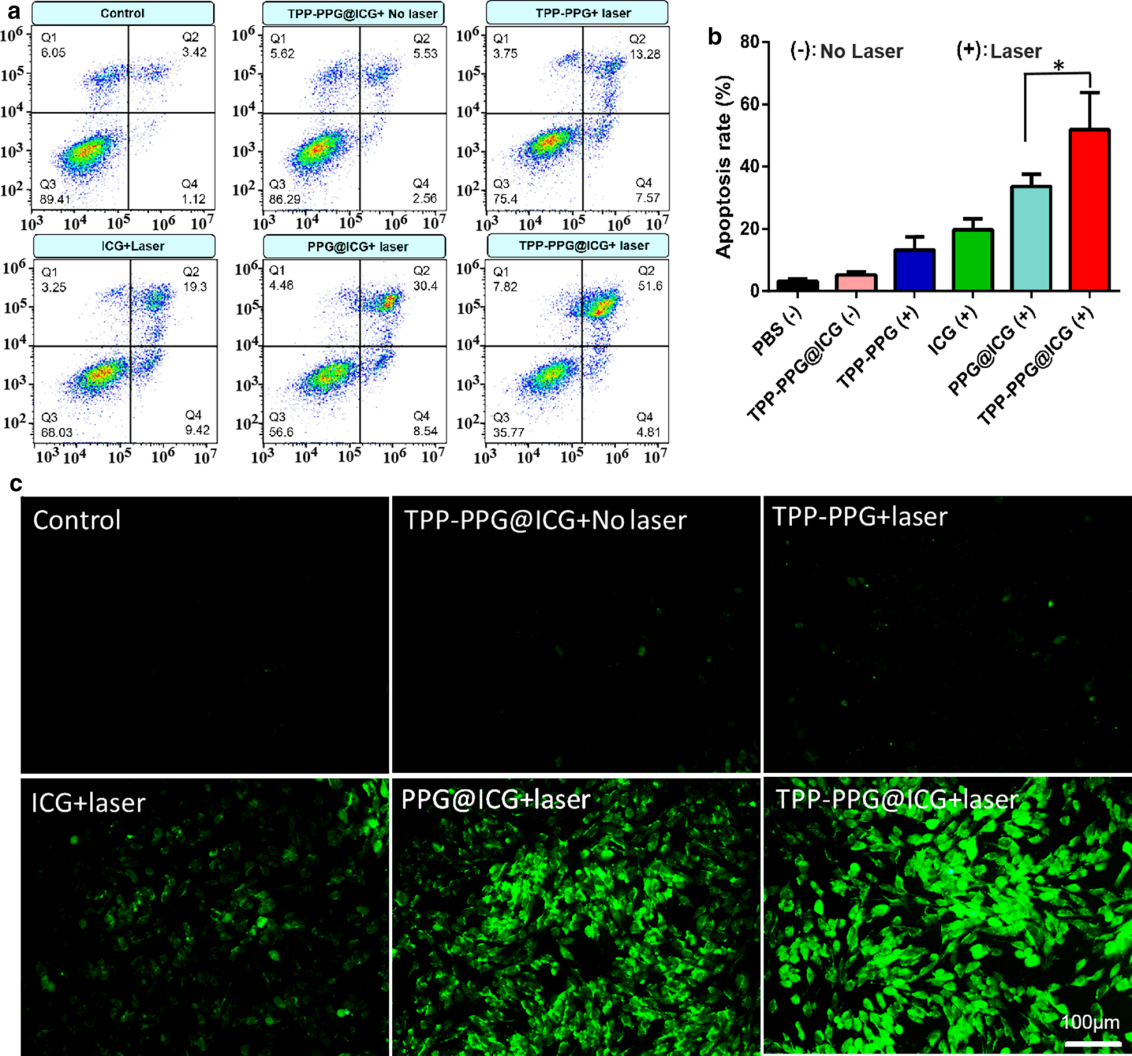
Cellular mechanism of the synergistic phototherapy of TPP-PPG@ICG. MG63/Dox cells were treated with the PBS, TPP-PPG, ICG, PPG@ICG, or TPP-PPG@ICG (ICG equivalent, 15 μM) with laser irradiation, or with TPP-PPG@ICG without laser irradiation. The rate of apoptosis (**a**, **b**) and **c** ROS production was measured using flow cytometry and FL microscopy after incubation with annexin V-FITC/PI or DCFH-DA, respectively. P values in **b** were calculated using student’s t-test (*p < 0.01)

Apoptosis has been reported to be the main mechanism of phototherapy [46]. Flow cytometry was used to evaluate the rate of apoptosis by assessing the fluorescein isothiocyanate (FITC)-labeled annexin V (annexin V-FITC) positivity of MG63/Dox cells, induced by TPP-PPG@ICG. Relatively few MG63/Dox cells treated with (i) TPP-PPG@ICG without irradiation, (ii) TPP-PPG plus irradiation, or (iii) ICG plus irradiation were positive for annexin V-FITC staining. In contrast, MG63/Dox cells exposed to laser irradiation at 808 nm after TPP-PPG@ICG treatment exhibited a significantly higher proportion of annexin V-FITC-positive cells (Fig. 5a, b). These results demonstrated that MG63/Dox cell death was mediated primarily via apoptosis.

PDT-induced apoptosis was mainly induced by intracellular ROS generation. Thus, to investigate the mechanism through which TPP-PPG@ICG killed the drug-resistant OS cells, we examined ROS generation in MG63/Dox cells irradiated with TPP-PPG@ICG using DCFH-DA. Intracellular ROS generation, which activates apoptosis, was recognized as the main cytotoxic mechanism associated with PDT treatment. DCFH-DA, a fluorimetric probe mainly used for oxidative stress measurements, is a non-fluorescent compound that can be transformed to a fluorescent compound (DCF) via an ROS-mediated oxidation reaction [47]. Accordingly, the intracellular FL intensity was measured to evaluate ROS generation using confocal FL microscopy. Green FL revealed the extent of intracellular ROS generation. Our results indicated that TPP-PPG@ICG generated negligible intracellular ROS without laser irradiation. After laser irradiation, intracellular ROS was generated to different extents in TPP-PPG-treated, ICG-treated, PPG@ICG-treated, and TPP-PPG@ICG-treated cells, among which, ROS generation was particularly robust in the TPP-PPG@ICG-treated cells, demonstrating the occurrence of mitochondria-targeted ROS production (Fig. 5c).

**Fig. 6:**
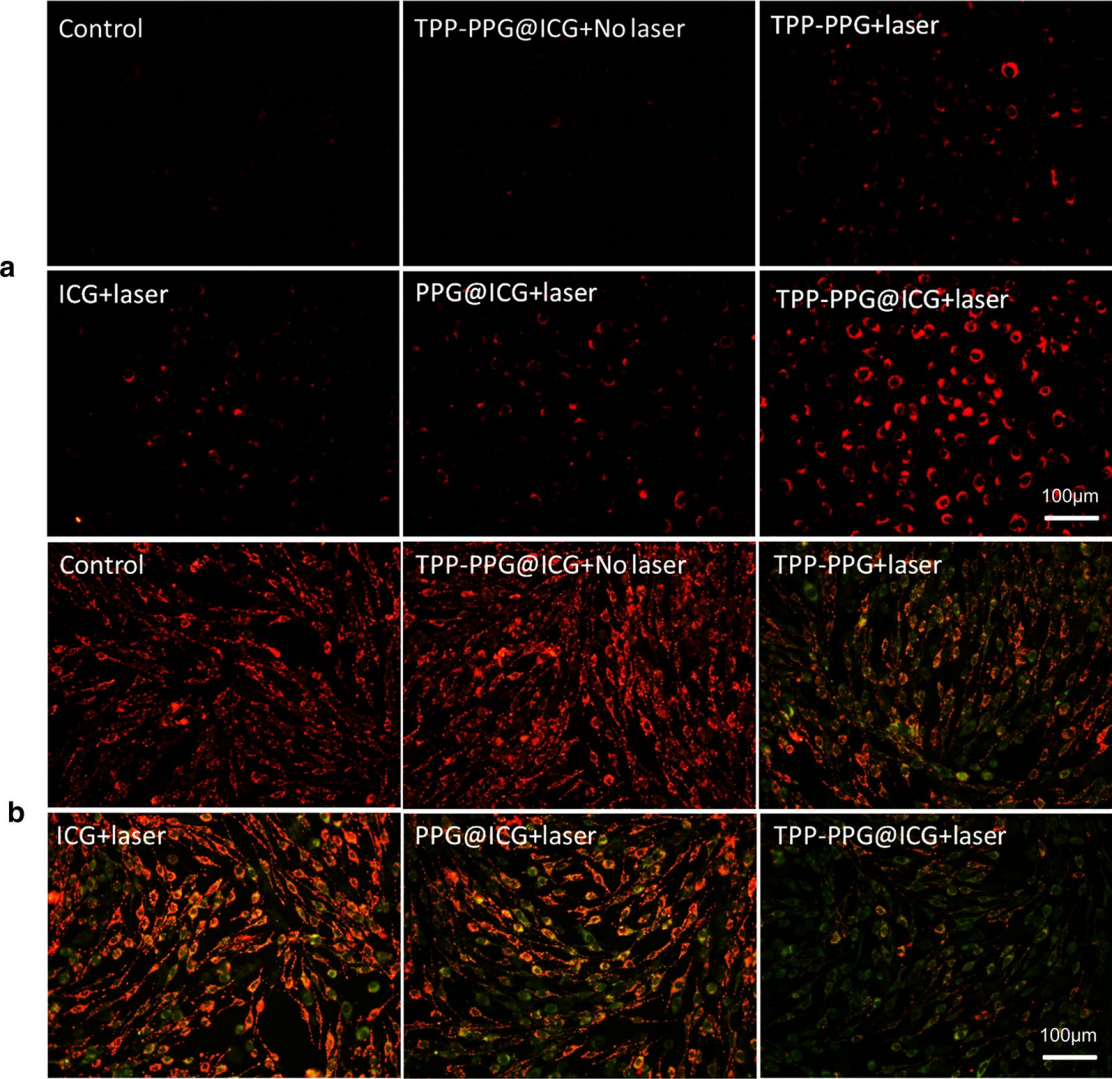
Cellular mechanism of synergistic phototherapy with TPP-PPG@ICG. MG63/Dox cells were treated with PBS, TPP-PPG, ICG, PPG@ICG, or TPP-PPG@ICG (ICG equivalent, 15 μM) with laser irradiation, or they were treated with TPP-PPG@ICG without laser irradiation. **a** Mitochondrial superoxide production and **b** mitochondrial membrane potentials were measured using MitoSOX Red and JC-1, respectively

As mentioned above, mitochondria are the central initiators of the intrinsic-apoptosis pathway, which makes them susceptible to photocytotoxicity. As TPP-PPG@ICG has been hypothesized to target the mitochondria, we focused on a potential synergistic phototherapeutic effect of TPP-PPG@ICG on the mitochondria. We evaluated superoxide production in the mitochondria of MG63/Dox cells using the mitochondrial superoxide indicator, MitoSOX Red. As shown in Fig. 6a, MitoSOX Red exhibited red FL in TPP-PPG@ICG-treated cells after 5 min of photoirradiation, whereas no appreciable red FL was observed in TPP-PPG@ICG-treated cells without photoirradiation, and weak red FL was detected in TPP-PPG-treated, ICG-treated, or PPG@ICG-treated cells after photoirradiation. These findings indicated that ICG and NGO were delivered simultaneously to the mitochondria in the composite nanostructures and exerted a synergistic effect. Thus, TPP-PPG@ICG may cause photodamage to mitochondria and perturb their functions.

The mitochondrial membrane potential reflects the functional status of mitochondria, which has been hypothesized to be associated with tumorigenicity, cell differentiation, and malignancy [9]. A previous report showed that cancer cells possess higher mitochondrial membrane potentials than normal cells [48]. Therefore, cancer cells exhibiting a markedly reduced mitochondrial membrane potential show suppressed ATP production, slower proliferation, and severe impairment of tumorigenicity [49, 50]. In this study, the mitochondrial membrane potential was studied by performing 5,5′,6,6′-tetrachloro-1,1′,3,3′-tetraethylbenzimidazolocarbocyanine iodide (JC-1) assays. As a cationic dye, JC-1 can accumulate in the mitochondria to form aggregates (red FL) in the presence of a high mitochondrial membrane potential, or it may disperse in the cytoplasm as monomers (green FL) if the mitochondrial membrane potential is reduced [51]. As shown in Fig. 6b, green FL was observed in TPP-PPG@ICG-treated cells after 5 min of photoirradiation, whereas appreciable red FL appeared in TPP-PPG@ICG-treated cells without photoirradiation, and weak green FL appeared in TPP-PPG, ICG, or PPG@ICG-treated cells after photoirradiation. These variations confirmed the mitochondrial targeting of TPP-conjugated ICG and NGO (with irradiation) and their ability to synergistically disrupt the mitochondrial function. Thus, TPP-PPG@ICG treatment may photodamage the mitochondria and affect their function. It is well known that mitochondria are the major functional components of ATP production [52]. Next, we continued to explore if the synergistic phototherapeutic could influence the ATP production in MG63/Dox cells after irradiation. As shown in Fig. 2c, compared with other groups, the intracellular ATP concentration decreased tremendously in TPP-PPG@ICG-treated cells after 5 min of photoirradiation. These results demonstrated that TPP-PPG@ICG plus irradiation could inhibit the cellular ATP production and disturb the cancer cell energy supply, which could assist to inhibit the ATP-dependent efflux of anticancer drugs. Hence, the greater TPP-PPG@ICG could enter the MG63/Dox cells to exert effect. As a result, the cancer cells were more sensitive to the damage induced by synergistic phototherapeutic.

Herein, based on the results of cellular uptake behavior, intracellular localization, intracellular ROS producing ability, ATP level, and so on, we hypothesize that the primary mechanism of the synergistic phototherapy of TPP-PPG@ICG. Aided by TPP-PPG, the significantly enhanced ICG delivery to the cell mitochondria was observed. Then, under the irradiation, ROS and hyperthermia were generated to perturb the mitochondrial membrane potentials, reduce the ATP production. Eventually, cell was death mediated by apoptosis.

### In vivo tumor targeted NIR imaging and synergistic phototherapy

**Fig. 7:**
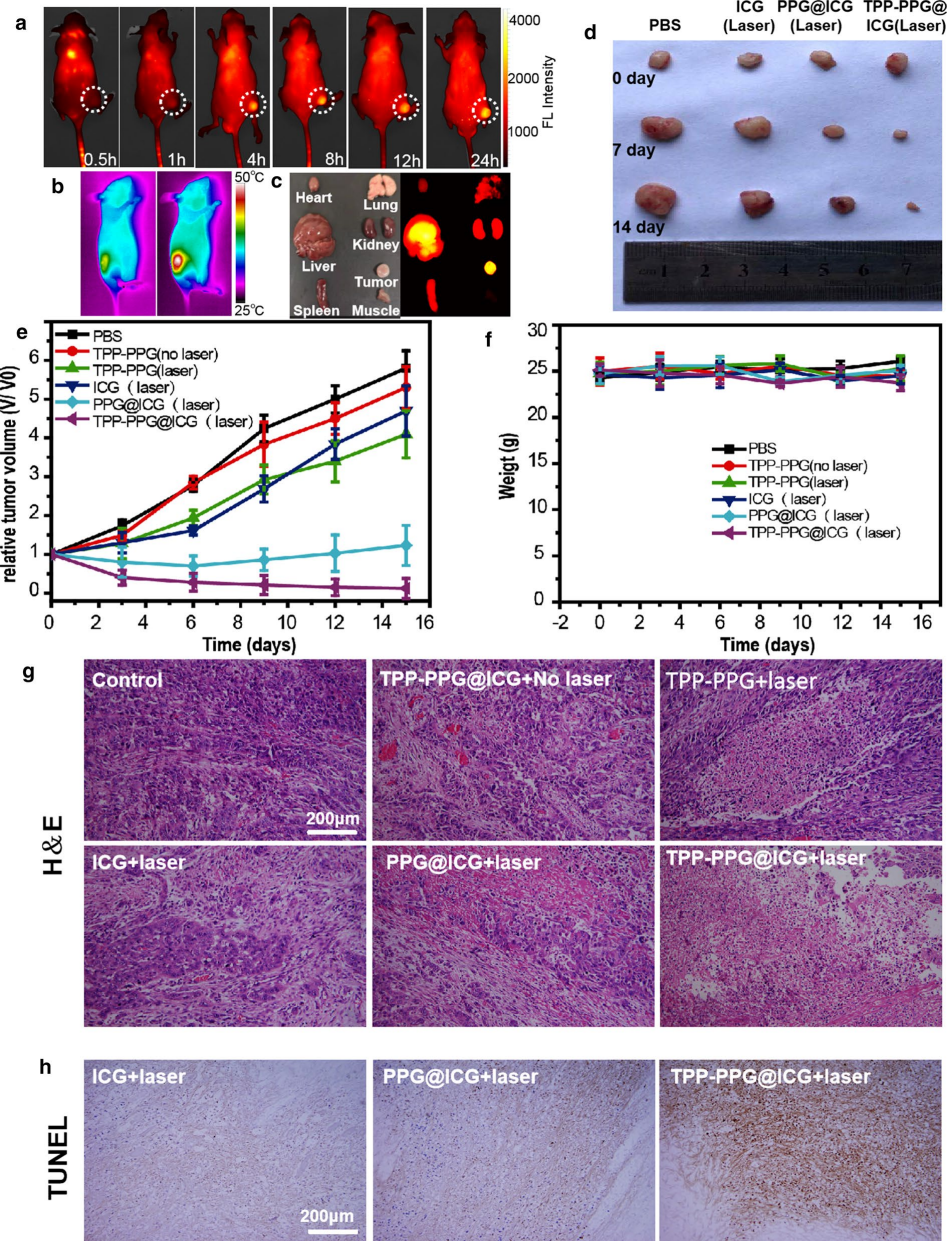
In vivo synergistic phototherapy of TPP-PPG@ICG on MG63/Dox tumor xenografts. **a** NIR FL imaging of MG63/Dox tumor xenografts exposed to 808-nm laser irradiation (0.6 W/cm^2^, 5 min), 0.5, 1, 4, 8, 12 and 24 h after tail vein injection of TPP-PPG@ICG, and **c** the dissected organs from the MG63/Dox tumor-bearing mice: the tumor, the lungs, the liver, the spleen, the kidneys, the heart and the muscle). **b** IR thermal imaging of MG63/Dox tumor xenografts exposed to 808-nm laser irradiation (0.6 W/cm^2^, 5 min), 24 h after tail vein injection with PBS and TPP-PPG@ICG. **d** Photographs of representative tumors resected from different groups on days 0, 7, and 14, as indicated. **e** Tumor volumes of MG63/Dox tumor xenografts, **f** body weights of different groups of mice, and **g** H&E and **h** TUNEL staining of tumor sections of different groups. TUNEL-staining images for the control, TPP-PPG@ICG + no laser, and TPP-PPG + laser groups are shown in Additional file 1: Fig. S9)

Based on the FL imaging in vivo and synergistic phototherapy in vitro, we evaluated TPP-PPG@ICG for imaging-guided drug-resistant OS phototherapy in vivo. The in vivo PTT efficiency of TPP-PPG@ICG was investigated in male, nude mice bearing MG63/Dox tumor xenografts. TPP-PPG@ICG (10 mg/kg) was intravenously injected into the tumor-bearing naked mice. In vivo NIR fluorescence imaging was taken from 0 to 24 h after injection. As exhibited in Fig. 7a, the fluorescence intensity at the implanted tumors increased in time-dependent manner and strong fluorescence intensity was observed at 24 h. Besides, dissected organs from the MG63/Dox tumor-bearing mice showed strong FL intensities in the tumors, but not in other organs, and their margins were clearly observable (Fig. 7c).

TPP-PPG@ICG (10 mg/kg) was intravenously injected into the tumor-bearing mice. At 24 h post-injection, in vivo IR thermal imaging was performed after a 5-min laser irradiation at 808 nm. When the entire tumor tissue was under irradiation, temperature changes in the tumor xenografts were recorded using an IR thermal camera. As shown in Fig. 7b and Additional file 1: Fig. S7, after 5 min of NIR laser irradiation, the temperature of the PBS-treated tumors increased from 34 °C to 42–44 °C. However, the temperature of tumors treated with the TPP-PPG@ICG increased to 60–62 °C, which was sufficient to kill malignant cells. In contrast, no apparent temperature increase was observed in other body parts of the mice. NIR imaging and the preferential accumulation of TPP-PPG@ICG in tumors indicated that this approach can be used for localizing the tumor margin and administering precise real-time imaged-guided cancer phototherapy.

In addition, the relative growth rate of the MG63/Dox tumors was monitored after injection. Tumors treated with PBS or TPP-PPG@ICG without irradiation grew rapidly. The growth rate of tumors in the TPP-PPG-treated and ICG-treated groups irradiated with the 808-nm laser was slightly inhibited, suggesting partially suppressed tumor growth. However, the tumor volumes increased 3 days after irradiation. In addition, the tumor growth rate in the PPG@ICG-treated group exposed to 808-nm laser irradiation was further inhibited, which suggested that an enhanced inhibitory effect on tumor growth occurred due to the synergistic effect of PDT and PTT. Nevertheless, tumor volumes still increased from 6 days after irradiation. In contrast, tumor growth in the TPP-PPG@ICG-treated irradiation group was significantly inhibited, which suggested that mitochondrial targeting may considerably improve the efficacy of synergistic phototherapy (Fig. 7d, e). Mitochondria are sensitive to ROS and hyperthermia; hence, phototherapy-mediated damages can rapidly perturb the mitochondrial function, change the mitochondrial membrane potential, reduce ATP production, and finally induce apoptosis in tumor cells. In addition to suppressing ATP production, mitochondria-targeted synergistic phototherapy can effectively circumvent drug resistance by inhibiting the P-gP-dependent unidirectional expulsion of anticancer drugs. Therefore, mitochondria-targeted synergistic phototherapy acted as an efficient strategy against drug-resistant OS. Body weights were monitored during treatment for all treated groups. No significant differences were observed among the groups. (Fig. 7f).

To further study the phototherapeutic effects of TPP-PPG@ICG, hematoxylin and eosin (H&E) and terminal deoxynucleotidyl transferase dUTP nick end labeling (TUNEL) staining of the tumor sections were performed at 24 h post-irradiation. Without irradiation, the PBS- and TPP-PPG@ICG-treated groups exhibited no tumor cell apoptosis or necrosis in the histological sections. Following irradiation, some sporadic necrosis was observed in the PPG@ICG-treated, which was more obvious than in the TPP-PPG-treated groups and ICG-treated group. In contrast, the most cancer cell apoptosis and necrosis occurred in the TPP-PPG@ICG-treated group, following irradiation. These findings indicated that treatment with TPP-PPG@ICG plus a single NIR laser (808 nm) synergistically enhanced the effects of PDT and PTT (Fig. 7g, h and Additional file 1: Fig. S8 ).

Furthermore, major organs, including the heart, liver, spleen, lungs, and kidneys, were collected from mice administered with TPP-PPG@ICG, for H&E staining to assess the side effect. The results showed that, compared to the degree of injury observed in the control group, injury was negligible after treatment with TPP-PPG@ICG (Additional file 1: Fig. S9). Otherwise, in order to further investigate the biosafety of TPP-PPG@ICG, the blood biochemistry was systematically performed. Alanine aminotransferase (ALT), aspartate aminotransferase (AST), and alkaline phosphatase (ALP) were selected to evaluate the liver function, while creatinine and urea nitrogen were measured to evaluate the kidney function. Meanwhile, the following important hematological markers: white blood cells (WBC), red blood cells (RBC), hematocrit (HCT), mean corpuscular volume (MCV), hemoglobin (HGB), platelets (PLT), mean corpuscular hemoglobin (MCH), and mean corpuscular hemoglobin concentration (MCHC) were chosen to evaluate the potential toxicity of TPP-PPG@ICG. As exhibited in Additional file 1: Tables S1 and S2, all the parameters in the TPP-PPG@ICG treated groups appear to be normal compared with the control groups, and within the reference normal ranges at day 30. Thus, the results exhibited that TPP-PPG@ICG showed high efficacy in tumor-targeted phototherapy with minimal side effects.

## Conclusions

In summary, we developed a new NGO derivative (TPP-PPG@ICG), which targets the mitochondria for FL image-guided synergistic phototherapy of drug-resistant OS. Owing to its preferential tumor accumulation, suitability for NIR imaging, and mitochondria-targeting ability, the TPP-PPG@ICG nanocomposite showed maximal phototherapeutic efficacy and minimal side effects. The PEG- and BPEI-functionalized NGO facilitated ICG dispersion and cellular uptake, whereas the mitochondria-targeted TPP simultaneously enhanced PDT/PTT cancer treatment and suppressed ATP production, which can serve as another effective strategy for surmounting drug resistance. The in vitro and in vivo phototherapy effects of TPP-PPG@ICG were significantly improved, leading to superior tumor eradication in drug-resistant OS models. The further improved novel nanocomposite shows promise for applications in highly sensitive FL imaging and efficient drug-resistant OS phototherapy. Thus, we expect that it has potential for therapeutic efficacy in prospective clinical trials.

## Supplementary Information

**Additional file 1: Figure S1.** The DLS analysis of the TPP-PPG and TPP-PPG@ICG. **Figure S2.** (a) The stabilities of TPP-PPG@ICG in PBS (A), in DMEM (B) and in PBS + 10% FBS (C). The pictures of the ultrafiltration tube and the filtrate after centrifugation at 5000 rpm min^−1^ for 10 min. (b) The UV–vis absorption spectra of the TPP-PPG@ICG incubation 24 h with 50% isopropanol, PBS, DMEM and PBS + 10% FBS after the appropriate cleaning procedures performed. **Figure S3.** The DLS change curve of TPP-PPG@ICG in PBS or PBS + 10% FBS. **Figure S4.** The effect of weight ratio of ICG and TPP-PPG on the ICG loading efficiency. **Figure S5.** The calibration curve of ICG in PBS with absorption at 790 nm. **Figure S6.** The MG63/Dox cells showed resistance to Dox. **Figure S7.** The temperature variation of tumors after laser exposure. **Figure S8.** TUNEL staining of tumor sections of control group,  TPP-PPG@ICG + No laser group and TPP-PPG + laser group. **Figure S9.** Negligible injury to the heart, the liver, the spleen, the lungs, and the kidneys after treatment with the TPP-PPG@ICG. **Table S1.** Comparison between PBS and TPP-PPG@ICG groups of treatment on whole blood cell count. **Table S2.** Comparison between PBS and TPP-PPG@ICG groups of treatment on liver function and kidney function indicators.

## Data Availability

All data generated or analyzed during this study are included in this article and its Additional file.

## Abbreviations

OS: Osteosarcoma
TPP: (4-Carboxybutyl) triphenyl phosphonium bromide
ATP: Adenosine triphosphate
P-gP: P-glycoprotein
PDT: Photodynamic therapy
PTT: Photothermal therapy
PS: Photosensitizer
ROS: Reactive oxygen species
NIR: Near-infrared
FL: Fluorescence
ICG: Indocyanine green
PEG: Polyethylene glycol
PEI: Polyethyleneimine
EDC: (3-Dimethylamino propyl-*N*′-ethylcarbodiimide) hydrochloride (EDC·HCl)
NHS: *N*-Hydroxysuccinimide
SOSG: Singlet oxygen sensor green
PI: Propidium iodide
JC-1: 5,5′,6,6′-tetrachloro-1,1′,3,3′-tetraethylbenzimidazolocarbocyanine iodide
CCK-8: Cell Counting Kit-8
FT-IR: Fourier transform infrared spectroscopy
AFM: Atomic force microscope
UV–vis–NIR: Ultraviolet–visible–infrared
CLSM: Confocal laser-scanning microscope
FL: Fluorescence
DCFH-DA: 2′,7′-dichlorodihydrofluorescein diacetate
H&E: Hematoxylin and eosin
TUNEL: Terminal deoxynucleotidyl transferase dUTP nick end labeling staining
ALT: Alanine aminotransferase
AST: Aspartate aminotransferase
ALP: Alkaline phosphatase
WBC: White blood cells
RBC: Red blood cells
HCT: Hematocrit
MCV: Mean corpuscular volume
HGB: Hemoglobin
PLT: Platelets
MCH: Mean corpuscular hemoglobin
MCHC: Mean corpuscular hemoglobin concentration
