# Supplementary material for: Mitochondria-targeting graphene oxide nanocomposites for fluorescence imaging-guided synergistic phototherapy of drug-resistant osteosarcoma
Source: J Nanobiotechnology. 2021 Mar 19;19:79. doi: 10.1186/s12951-021-00831-6 (PMC7980640; doi:10.1186/s12951-021-00831-6)
Supplement: Supplementary file 1 — Additional file 1: Figure S1. The DLS analysis of the TPP-PPG and TPP-PPG@ICG. Figure S2. (a) The stabilities of TPP-PPG@ICG in PBS (A), in DMEM (B) and in PBS + 10% FBS (C). The pictures of the ultrafiltration tube and the filtrate after centrifugation at 5000 rpm min−1 for 10 min. (b) The UV–vis absorption spectra of the TPP-PPG@ICG incubation 24 h with 50% isopropanol, PBS, DMEM and PBS + 10% FBS after the appropriate cleaning procedures performed. Figure S3. The DLS change curve of TPP-PPG@ICG in PBS or PBS + 10% FBS. Figure S4. The effect of weight ratio of ICG and TPP-PPG on the ICG loading efficiency. Figure S5. The calibration curve of ICG in PBS with absorption at 790 nm. Figure S6. The MG63/Dox cells showed resistance to Dox. Figure S7. The temperature variation of tumors after laser exposure. Figure S8. TUNEL staining of tumor sections of control group, TPP-PPG@ICG + No laser group and TPP-PPG + laser group. Figure S9. Negligible injury to the heart, the liver, the spleen, the lungs, and the kidneys after treatment with the TPP-PPG@ICG. Table S1. Comparison between PBS and TPP-PPG@ICG groups of treatment on whole blood cell count. Table S2. Comparison between PBS and TPP-PPG@ICG groups of treatment on liver function and kidney function indicators. [file 12951_2021_831_MOESM1_ESM.docx]

**Supplementary information**

**Mitochondria-targeting graphene oxide nanocomposites for fluorescence imaging-guided synergistic phototherapy of drug-resistant osteosarcoma**

Wei-Nan Zeng ^1,2^, Qiu-Ping Yu ^3^, Duan Wang ^1^, Jun-Li Liu ^2^, Qing-Jun Yang ^2, *^, Zong-Ke Zhou ^1, *^, Yi-Ping Zeng ^2, *^

^1^ Department of Orthopedics, West China Hospital/West China School of Medicine, Sichuan University, Chengdu, 610041, China

^2^ Department of Orthopedics, Chongqing General Hospital, University of Chinese Academy of Sciences, Chongqing, 400014, China.

^3^ Health Management Center, West China Hospital/West China School of Medicine, Sichuan University, Chengdu, 610041, China

**Corresponding author.
E-mail address: zongkehx@163.com (Z.K. Zhou);* [*qingjunyang_cgh@163.com (Q.J*](mailto:qingjunyang_cgh@163.com%20(Q.J)*. Yang); zyp860625@126.com (Y.P. Zeng)*


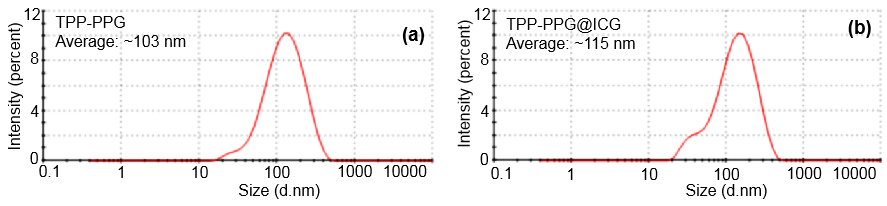


**Fig. S1**. The DLS analysis of the TPP-PPG and TPP-PPG@ICG


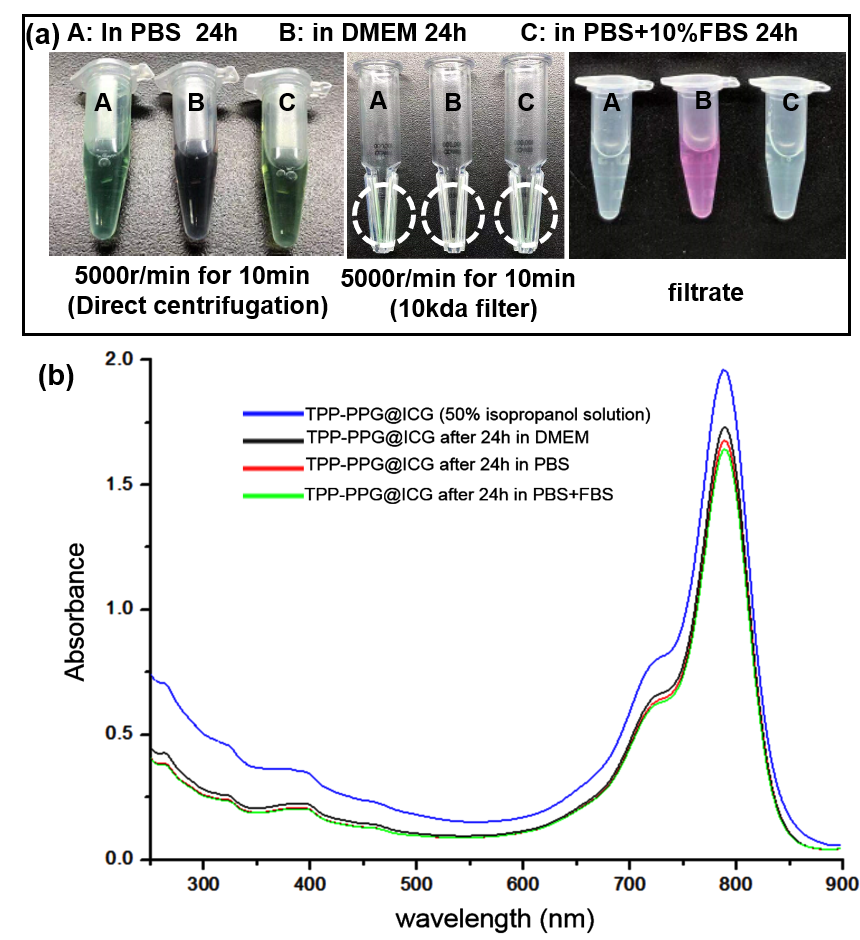


**Fig. S2**. (a) The stabilities of TPP-PPG@ICG in PBS (A), in DMEM(B) and in PBS+10% FBS (C), no precipitation was observed after centrifugation at 8000 rpm min^-1^ for 10 min. The pictures of the ultrafiltration tube and the filtrate after centrifugation at 5000 rpm min^-1^ for 10min. (b) The UV−vis absorption spectra of the TPP-PPG@ICG incubation 24h with 50% isopropanol, PBS, DMEM and PBS+10% FBS after the appropriate cleaning procedures performed.


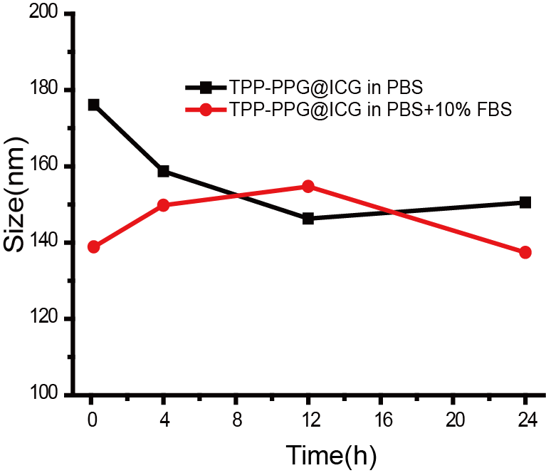


**Fig. S3.** The DLS change curve of TPP-PPG@ICG in PBS or PBS + 10% FBS.


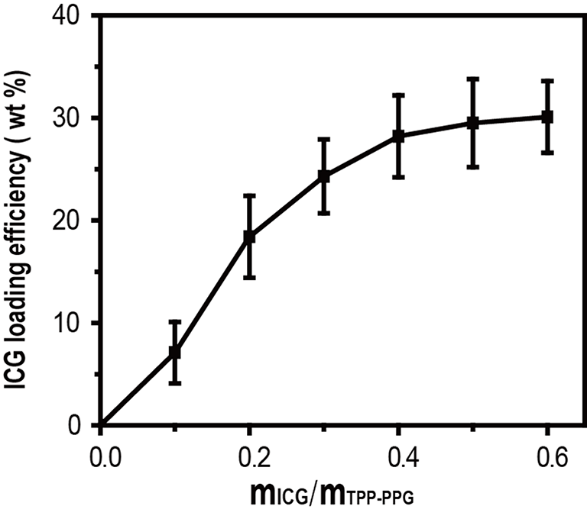


**Fig. S4.** The effect of weight ratio of ICG and TPP-PPG on the ICG loading efficiency.

**
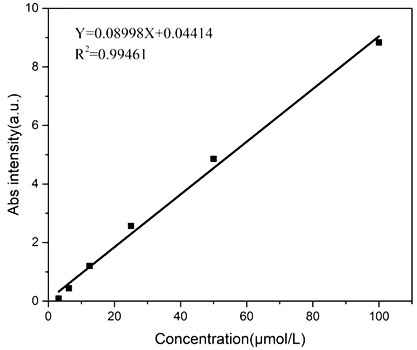
**

**Fig. S5.** The calibration curve of ICG in PBS with absorption at 790 nm.


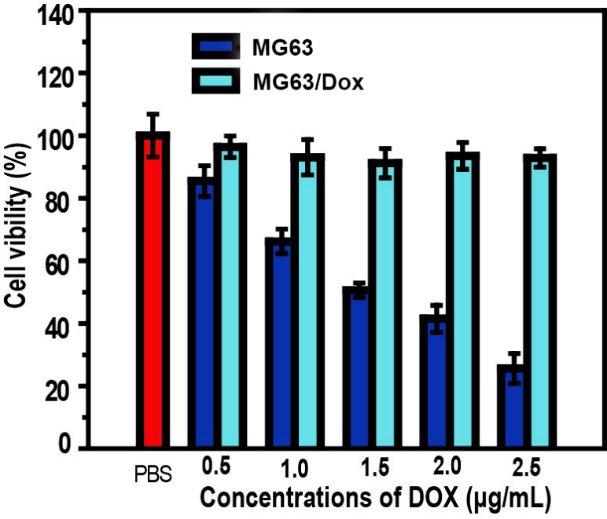


**Fig. S6.** The MG63/Dox cells showed resistance to Dox.

**
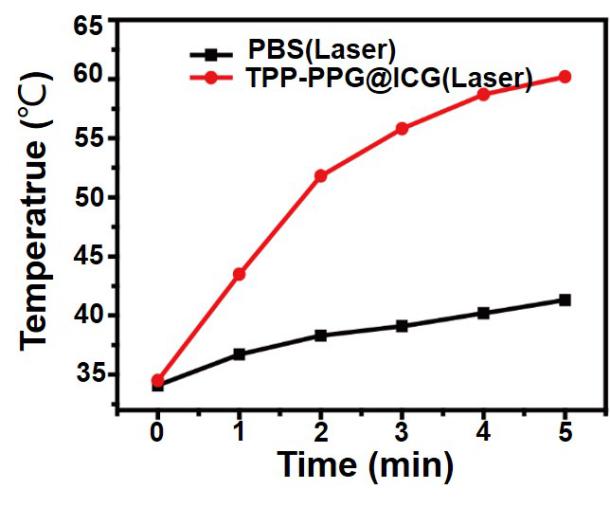
**

**Fig. S7.** The temperature variation of tumors after laser exposure.


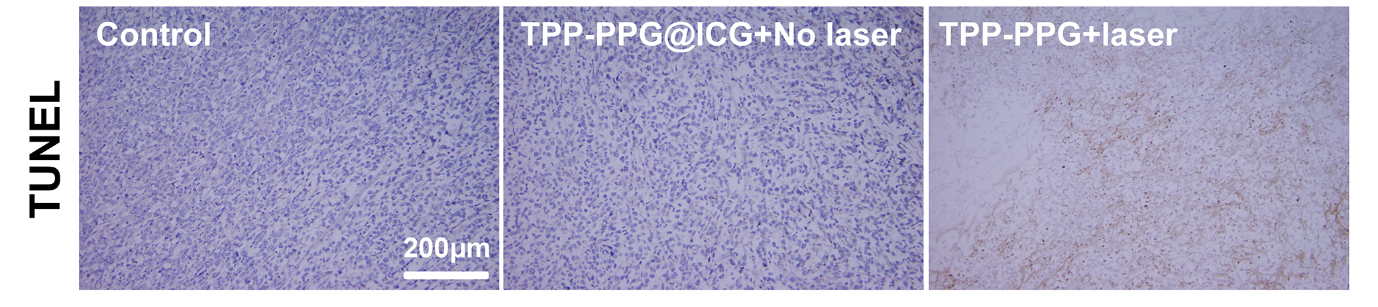


**Fig. S8.** TUNEL staining of tumor sections of control group, TPP-PPG@ICG+No laser group and TPP-PPG+laser. group


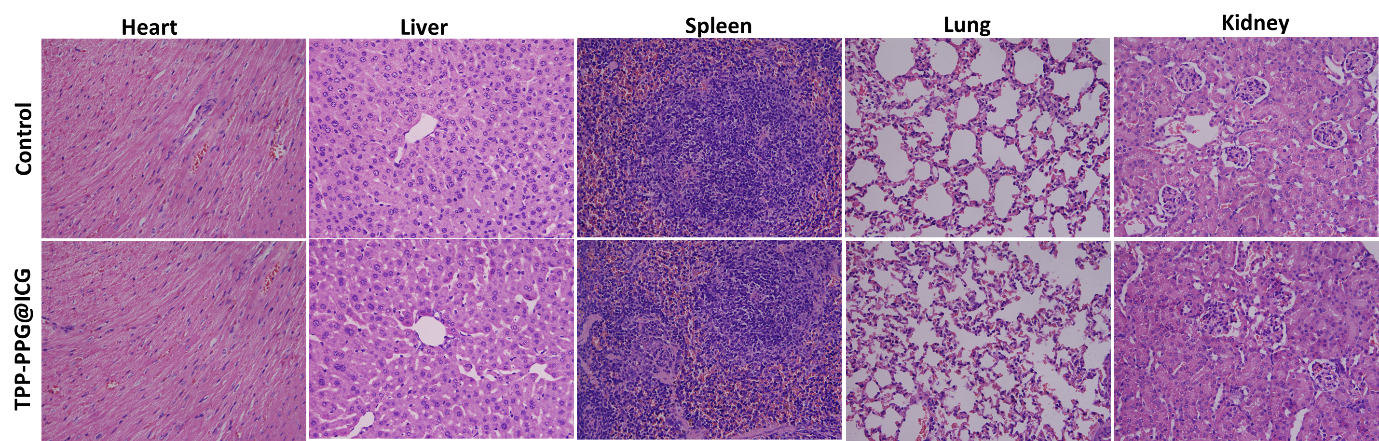


**Fig. S9.** Negligible injury to the heart, the liver, the spleen, the lungs, and the kidneys after treatment with the TPP-PPG@ICG.

**Table 1** Comparison between PBS and TPP-PPG@ICG groups of treatment on whole blood cell count.

| Group | WBC (10^9/L) | RBC (10^12/L) | HGB (g/L) | HCT (%) | MCV (FL) | MCH (pg) | MCHC (g/L) | PLT (10^9/L) |
| --- | --- | --- | --- | --- | --- | --- | --- | --- |
| PBS | 5.116± 0.567 | 9.596± 0.370 | 159± 5.34 | 51.56± 0.937 | 51.36± 1.379 | 17.72± 0.828 | 338.6± 10.41 | 415± 15.03 |
| TPP-PPG@ICG | 5.592± 0.472 | 10.356± 0.298 | 156.6± 5.94 | 54.38± 1.642 | 50.96± 3.111 | 18.54± 1.026 | 341.2± 13.24 | 421.4± 23.09 |

Values are mean±SEM

**Table 2** Comparison between PBS and TPP-PPG@ICG groups of treatment on liver function and kidney function indicators

| Group | ALT (IU/L) | ALP (IU/L) | AST(IU/L) | CREA(μmol/L) | BUN (mmol/L) |
| --- | --- | --- | --- | --- | --- |
| PBS | 55.68±5.634 | 143.24±8.452 | 155.6±13.212 | 47.28±3.237 | 8.735±0.392 |
| TPP-PPG@ICG | 46.68±6.247 | 153.65±7.425 | 143.2±10.256 | 52.76±2.832 | 7.478±0.582 |

Values are mean±SEM
